# Hepatic Lipidomics and Molecular Imaging in a Murine Non-Alcoholic Fatty Liver Disease Model: Insights into Molecular Mechanisms

**DOI:** 10.3390/biom10091275

**Published:** 2020-09-03

**Authors:** Ricardo Rodríguez-Calvo, Sara Samino, Josefa Girona, Neus Martínez-Micaelo, Pere Ràfols, María García-Altares, Sandra Guaita-Esteruelas, Alexandra Junza, Mercedes Heras, Oscar Yanes, Xavier Correig, Lluis Masana

**Affiliations:** 1 Vascular Medicine and Metabolism Unit, Research Unit on Lipids and Atherosclerosis, “Sant Joan” University Hospital, Universitat Rovira i Virgili, Institut de Investigació Sanitaria Pere Virgili (IISPV), 43204 Reus, Spain; josefa.girona@urv.cat (J.G.); neus.martinez@urv.cat (N.M.-M.); sandra.guaita@urv.cat (S.G.-E.); mercedes.heras@urv.cat (M.H.); 2 Spanish Biomedical Research Centre in Diabetes and Associated Metabolic Disorders (CIBERDEM), Institute of Health Carlos III, 28029 Madrid, Spain; sara.samino@estudiants.urv.cat (S.S.); pere.rafols@urv.cat (P.R.); maria.garcia-altares@urv.cat (M.G.-A.); alexandra.junza@urv.cat (A.J.); oscar.yanes@urv.cat (O.Y.); xavier.correig@urv.cat (X.C.); 3 Metabolomics Platform, Department of Electronic Engineering (DEEEA), Universitat Rovira i Virgili, 43007 Tarragona, Spain

**Keywords:** NAFLD, lipidome, adipokines, inflammation, liver triglycerides, FABP4

## Abstract

An imbalance between hepatic fatty acid uptake and removal results in ectopic fat accumulation, which leads to non-alcoholic fatty liver disease (NAFLD). The amount and type of accumulated triglycerides seem to play roles in NAFLD progression; however, a complete understanding of how triglycerides contribute to NAFLD evolution is lacking. Our aim was to evaluate triglyceride accumulation in NAFLD in a murine model and its associations with molecular mechanisms involved in liver damage and adipose tissue-liver cross talk by employing lipidomic and molecular imaging techniques. C57BL/6J mice fed a high-fat diet (HFD) for 12 weeks were used as a NAFLD model. Standard-diet (STD)-fed animals were used as controls. Standard liver pathology was assessed using conventional techniques. The liver lipidome was analyzed by liquid chromatography–mass spectrometry (LC–MS) and laser desorption/ionization–mass spectrometry (LDI–MS) tissue imaging. Liver triglycerides were identified by MS/MS. The transcriptome of genes involved in intracellular lipid metabolism and inflammation was assessed by RT-PCR. Plasma leptin, resistin, adiponectin, and FABP4 levels were determined using commercial kits. HFD-fed mice displayed increased liver lipid content. LC–MS analyses identified 14 triglyceride types that were upregulated in livers from HFD-fed animals. Among these 14 types, 10 were identified in liver cross sections by LDI–MS tissue imaging. The accumulation of these triglycerides was associated with the upregulation of lipogenesis and inflammatory genes and the downregulation of β-oxidation genes. Interestingly, the levels of plasma FABP4, but not of other adipokines, were positively associated with 8 of these triglycerides in HFD-fed mice but not in STD-fed mice. Our findings suggest a putative role of FABP4 in the liver-adipose tissue cross talk in NAFLD.

## 1. Introduction

Liver fat accumulation is considered the onset of non-alcoholic fatty liver disease (NAFLD), the main cause of chronic liver disease in the Western world [1,2,3]. NAFLD ranges in severity, with progression from non-alcoholic fatty liver (NAFL) to non-alcoholic steatohepatitis (NASH) [1,3,4]. If not corrected, NASH can progress to cirrhosis and hepatocellular carcinoma [5]. Based on clinical features, NAFLD (both NAFL and NASH) can be suspected in the presence of metabolic comorbidities. However, whereas NAFL can be diagnosed by imaging techniques, NASH diagnosis requires liver biopsy (for review, see [6]). Since most patients with NASH are asymptomatic and potentially advanced stages are difficult to detect, the identification of predictive biomarkers for disease progression is eagerly awaited.

Increased hepatic lipid content is the consequence of high free fatty acid delivery to the liver due to increased lipolysis in adipose tissue; typically, this increased lipolysis is mediated by insulin resistance in patients with obesity, metabolic syndrome, or type 2 diabetes, among other conditions [4,7]. Additionally, hyperinsulinemia induces hepatic *de novo* lipogenesis. Both mechanisms account for high fat acquisition in the liver that exceeds the capacity of the fat removal pathways and increased levels of very low-density lipoprotein (VLDL) secretion and mitochondrial fatty acid β-oxidation [4,7]. All these processes promote liver triglyceride accumulation [8,9]. The amount and type of triglyceride may influence NAFL-to-NASH progression by regulating cellular processes, such as cellular proliferation [10], mitochondrial dysfunction [11], inflammation [11,12], fibrogenesis [11], and endoplasmic reticulum (ER) stress-induced apoptosis [13]. However, a comprehensive understanding of the triglycerides involved in NAFLD progression is lacking. Since the progression from NAFL to NASH is not fully understood, the identification of the specific triglycerides involved in NAFL onset and their associations with the molecular mechanisms involved in lipid accumulation and liver injury would provide insight into the disease pathophysiology, leading to better clinical approaches.

Approximately 60% of the triglycerides accumulated in NAFLD originate from white adipose tissue [14,15]; therefore, this tissue is relevant to NAFLD pathogenesis. In fact, NAFLD prevalence correlates with the obesity rate and increases with the body mass index [16,17]. In particular, visceral obesity has been proposed as an important risk factor for the onset of NAFLD [17]. Apart from providing free fatty acids, adipocytes act as endocrine organs, secreting biologically active molecules termed adipokines [18], thereby taking part in the cross talk between adipose tissue and peripheral organs, including the liver [19]. Increasing evidence shows that some adipokines are involved in several processes related to the onset and progression of NAFLD [19]. Adiponectin has been found to be associated with hepatoprotective activities [20] and to be reduced under insulin-resistance conditions, including NASH [21]. In addition, leptin has been shown to be involved in NAFLD development and progression, participating in the liver fibrogenic response [22], and has been identified as a putative biomarker of disease progression [23]. The pathophysiological role of resistin in NAFLD is unclear. Whereas some studies have found no difference in resistin levels between NAFLD patients and control subjects [24], others have reported higher serum resistin levels in NAFLD patients [25], particularly those with advanced fibrosis [26]. Fatty acid-binding protein 4 (FABP4) is the main intracellular transporter of fatty acids in adipose tissue. It can be detected in plasma, and high levels of FABP4 are associated with metabolic disturbances [27]. FABP4 can induce intracellular lipid accumulation in peripheral tissues and a variety of cell types, including HepG2 liver cells [28]. Additionally, serum FABP4 levels have been found to be associated with NAFLD in both type 2 diabetic patients [29] and, apparently, healthy subjects [30]. The roles of these adipokines in determining liver triglyceride composition in NAFLD have not been explored.

In the present study, we performed a comprehensive lipid composition analysis and investigated the potential relationships between specific liver triglycerides and selected molecular pathways and adipokines in a murine model of NAFLD.

## 2. Research Design and Methods

### 2.1. Animal Model Experiment

Six-week-old C57BL/6J male mice were maintained on a standard light–dark cycle (12 h light–dark cycle) and temperature (21 ± 1 °C) conditions with ad libitum access to food and water. The animals were randomly distributed into two experimental groups (*n* = 10 each) and fed one of two diets for 12 weeks: a standard chow diet (STD: 10% kcal from fat; Panlab; Barcelona, Spain) and a high-fat diet (HFD: 60% kcal from fat; Panlab; Barcelona, Spain) [31,32]. After this period, the mice were euthanized, and the livers were immediately frozen in liquid nitrogen and stored at −80 °C. The experimental procedures conformed to the *Guide for the Care and Use of Laboratory Animals* published by the U.S. National Institutes of Health (NIH publication no. 85–23, revised 1996). All procedures were approved by the University Rovira i Virgili Bioethics Committee, as stated in Law 5/21 July 1995 passed by the Generalitat de Catalunya (Autonomous Government of Catalonia).

### 2.2. Biochemical Plasma Profile

After the animals were fasted for 4 h, blood samples were collected in heparin. The samples were analyzed for plasma levels of total cholesterol, high-density lipoprotein cholesterol (HDLc), glutamic oxaloacetic transaminase (GOT), and glutamate–pyruvate transaminase (GPT) (all from Spinreact, SA, Barcelona, Spain) by standardized colorimetric and enzymatic methods adapted to the Cobas Mira Plus Autoanalyser (Roche Diagnostics, Barcelona, Spain). The low-density lipoprotein cholesterol (LDLc) concentration was calculated using the Friedewäld formula, and the concentration of rich triglyceride particles (i.e., VLDLc, VLDL cholesterol) was determined by the formula total cholesterol - (HDLc + LDLc). Plasma levels of leptin, resistin, adiponectin (Milliplex^®^, Millipore; Billerica, MA, USA), and FABP4 (Bio Vendor Laboratory Medicine Inc., Brno, Czech Republic) were determined by commercial ELISA kits.

### 2.3. Liver Triglyceride Determination

Total lipids of liver homogenates were extracted according to the method of Bligh and Dyer [33], evaporated under N_2_, and redissolved in ethanol. Then triglyceride content was determined using a commercial kit (Spinreact, SA, Barcelona, Spain).

### 2.4. Standard Histological Analysis

Histological analyses were performed in 5 µm cross sections from frozen mouse livers. Liver architecture was assessed by hematoxylin and eosin staining. Lipid content was measured using Oil Red O stain following the protocol described by Mehlem et al. [34]. Fibrosis was measured by Sirius red staining. Photographs were obtained using an optical microscope (Olympus IX71, Barcelona, Spain).

### 2.5. RNA Preparation and Quantitative Real-Time Reverse Transcription–Polymerase Chain Reaction (RT-PCR) Analysis

mRNA levels were assessed by real-time RT-PCR as previously described [31]. Total RNA was isolated from livers using TRI Reagent (Sigma-Aldrich; Barcelona, Spain) according to the manufacturer’s recommendations. RNA integrity was determined by electrophoresis in agarose gel and was quantified by a NanoDrop 1000 spectrophotometer (Thermo Scientific; Madrid, Spain). Total RNA (1 μg) was reverse-transcribed using the PrimeScript RT Reagent Kit (Takara Bio; Saint-Germain-en-Laye, France). Levels of mRNA were assessed by real-time PCR on an ABI PRISM 7900 sequence detector (Applied Biosystems; CA, Beverly, USA). TaqMan gene expression assays-on-demand (IDT; Leuven, Belgium) were used for mouse peroxisome proliferator-activated receptor γ coactivator-1α (*Pgc1α*) (Mm.PT.58.17390716); mouse acyl-Coenzyme A (CoA) synthetase (*Acs*) (Mm.PT.58.29312796); mouse medium-chain acyl-CoA dehydrogenase (*Acadm*) (Mm.PT.58.9004416); mouse vey-long-chain acyl-CoA dehydrogenase (*Acadvl*) (Mm.PT.58.30103666); mouse acyl-CoA oxidase 1 (*Acox1*) (Mm.PT.58.5874703); mouse fatty acid synthase (*Fas*) (Mm.PT.58.14276063); mouse glycerol-3-phosphate acyltransferase (*Gpat1*) (Mm.PT.58.12176691); mouse diacylglycerol O-acyltransferase 2 (*Dgat2*) (Mm.PT.58.28629966); mouse collagen, type I, alpha 1 (*Col1a1*) (Mm.PT.58.7562513); mouse collagen, type I, alpha 2 (*Col1a2*) (Mm.PT.58.5206680); mouse chemokine (C-C motif) ligand 2 (*Ccl2*) (Mm.PT.58.42151692); and mouse tumor necrosis factor (*Tnf*) (Mm.PT.58.12575861). TATA-binding protein (*Tbp*) (Mm.PT.58.10867035) was used as the endogenous control [31].

### 2.6. Lipidomics

Lipids were extracted from 2 mg of lyophilized liver in methanol and 0.1% formic acid. After sample fragmentation by vortexing, immersion in liquid N_2_, and ultrasonication, 3 volumes of dichloromethane and 1 volume of water were added sequentially. Samples were incubated at 4 °C for 30 min and centrifuged (at 15,000 rpm for 15 min at 4 °C). The organic phase (lipidic) was collected for drying under a stream of nitrogen. Lipid pellets were resuspended in 150 µL of methanol/toluene (9:1) for liquid chromatography–mass spectrometry (LC–MS) analysis. Quality control (QC) samples consisting of pooled samples from each condition were injected at the beginning and periodically through the workflow.

Untargeted LC–MS analyses were performed using a UHPLC system (1200 series, Agilent Technologies) coupled with a 6550 ESI-QTOF MS (Agilent Technologies) operating in positive electrospray ionization (ESI+) mode. A total of 1 µL of sample was injected, and lipids were separated by reverse-phase chromatography with an Acquity UPLC C18-RP (ACQUITY UPLC BEH C18 1.7 µM, Waters). Mobile phase A was acetonitrile/water (60:40) (10 mM ammonium formate), and mobile phase B was isopropanol/acetonitrile (90:10) (10 mM ammonium formate). Solvent modifiers were used to enhance ionization and to improve the LC resolution in positive ionization mode. Separation was conducted under the following gradient: 0–2 min, 15–30% B; 2–2.5 min, 48% B; 2.5–11 min, 82% B; 11–11.5 min, 99% B; 11.5–12 min, 99% B; 12–12.1 min, 15% B; 12.1–15 min, 15% B. The ESI conditions were as follows: gas temperature, 150 °C; drying gas, 13 L min–1; nebulizer, 35 psig; fragmentor, 150 V; and skimmer, 65 V. The instrument was set to work over the *m/z* range from 50 to 1200 with an acquisition rate of 3 spectra/sec. For compound identification, MS/MS analyses were performed in targeted mode, and the instrument was set to acquire spectra over the *m/z* range from 700 to 900. The collision energy was fixed at 20 V. Lipid structures were identified by matching tandem MS spectra against reference standards in LIPID MAPS [35] and/or LipidBlast [36] and/or Metlin (https://metlin.scripps.edu) databases. The MS/MS spectra were identified by studying the pattern of triglyceride fragmentation due to losses of the different monoglycerides, which are part of the triglycerides.

LC–MS data were processed using XCMS [37] software (version 1.34.0) to detect and align mzRT features. XCMS analysis of these data provided a matrix containing the retention time, *m/z* value, and integrated the peak area of each feature for each sample. We constrained the initial number of features: QC samples were used to filter analytical variation as previously described [38], and only features above an intensity threshold of 5000 were retained for statistical analysis. Then, the data were normalized by dry weight.

### 2.7. Tissue Molecular Imaging

Tissue imaging by laser desorption/ionization–mass spectrometry (LDI–MS) imaging was performed as previously described [39,40]. Briefly, gold nanolayers were deposited over the 10 µm tissue sections using the ATC Orion 8-HV sputtering system (AJA International, N. Scituate, MA, USA), and LDI–MS tissue images were acquired using a MALDI-TOF UltrafleXtreme instrument with SmartBeam II Nd:YAG/355 nm laser from Bruker Daltonics. Data processing and visualization was based on the workflow described by Ràfols et al. [40] using the open-source software rMSI [41] and rMSIproc (https://github.com/prafols/rMSIproc). Tentative identification of triglycerides was based on the exact mass of their sodium adduct according to the Human Metabolome Database filtering metabolites with mass error Δ < 100 ppm and the results obtained from the LC–MS/MS experiments.

### 2.8. Statistical Analysis

Data are expressed as the mean ± standard error of the mean (SEM). Continuous variables were compared between groups using Student’s *t*-test. Correlations were evaluated using Pearson’s coefficients. Multiple linear regression analyses were used to test the influence of FABP4 on liver triglycerides. The linear regression results are expressed as β-coefficients with confidence intervals (CIs) and *R^2^* values. The association analyses were adjusted for weight, plasma non-esterified fatty acids (NEFAs), and plasma triglycerides. Statistical analyses were performed using SPSS software (IBM SPSS Statistics, version 22.0). Differences were considered statistically significant at *p* < 0.05.

## 3. Results

### 3.1. Characteristics of the HFD Mice Model

#### 3.1.1. HFD Mice Developed Obesity and Metabolic Disturbances

HFD-fed mice increased their body weight by 28%. The metabolic changes of HFD mice have been previously reported [31,32]. Additionally, HFD mice did not exhibit significant differences from STD mice in plasma total cholesterol (STD: 2.25 ± 0.10 mmol/L; HFD: 2.29 ± 0.14 mmol/L), HDLc (STD: 1.15 ± 0.07 mmol/L; HFD: 1.21 ± 0.13 mmol/L), and LDLc (STD: 0.91 ± 0.05 mmol/L; HFD: 0.94 ± 0.10 mmol/L); but they did in VLDLc (STD: 0.19 ± 0.02 mmol/L; HFD: 0.33 ± 0.03 mmol/L) (Figure S1).

#### 3.1.2. HFD-Induced Hepatic Steatosis in Mice

Hematoxylin and eosin staining showed increased vesicles in livers from HFD-fed animals relative to the numbers in livers from STD-fed mice (Figure S2A). In line with this observation, livers from animals fed the HFD showed greater lipid deposition than those from STD-fed animals, as demonstrated by Oil Red O staining (Figure S2B) and liver triglyceride content (Figure S2C). Liver fibrosis, as assessed by Sirius red staining, was not observed in livers from either experimental group (Figure S2D). Although a trend was observed, no significant increases in the plasma levels of liver injury hallmarks were found in the HFD-fed animals relative to the STD-fed animals, including GOT (Figure S2E), GPT (Figure S2F), and the GPT/GOT ratio (Figure S2G).

#### 3.1.3. Expression of Genes Involved in Fatty Acid Intracellular Lipid Metabolism and Inflammation Pathways

In accordance with biochemical and pathological data, relative to the STD mice, the HFD mice showed reduced expression of genes involved in fatty acid β-oxidation, such as *Pgc1α*, *Acs*, *Acadm*, *Acadvl*, and *Acox1* (Figure S3A), and increased expression of genes involved in the de novo synthesis of hepatic triglycerides, such as *Fas* and *Dgat2*; they showed no alteration in *Gpat1* expression (Figure S3B).

Although no significant differences between the groups were observed in liver damage or fibrosis, the expressions of *Col1a1* and *Col1a2* were induced in livers from animals fed with HFD (Figure S3C). Additionally, the expressions of inflammatory genes, such as *Ccl2* and *Tnf*, were higher in livers from HFD-fed animals than in livers from STD-fed animals (Figure S3D).

### 3.2. Liver Triglyceride Composition in HFD-Fed Mice Revealed by Lipidomics

**Figure 1:**
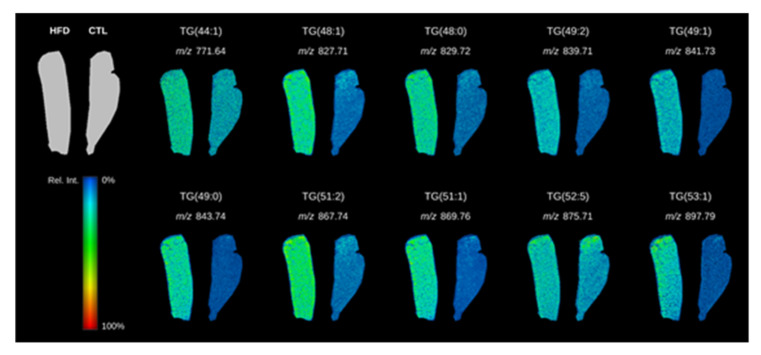
LDI–MS tissue imaging visualization of the distribution of triglycerides in liver cross sections from standard-diet (STD)- and high-fat-diet (HFD)-fed animals. A representative tissue image showing the abundance of 10 of the 14 triglycerides identified by LC-MS is shown. Green color denotes a higher abundance of a particular triglyceride, whereas blue denotes a lower abundance.

We analyzed liver lipid composition using LC–MS untargeted analyses. A total of 178 metabolites were found to be differentially expressed between STD- and HFD-fed animals. Among these metabolites, 58 were not identified. Sixty-one of the 120 metabolites putatively identified were classified as triglycerides. Thus, we focused on the identification of these molecules by MS/MS analysis performed in targeted mode. Fourteen of these triglycerides were upregulated in livers from HFD-fed animals compared with those from STD-fed animals: 43:1, 44:0, 44:1, 46:0, 47:0, 48:0, 48:1, 49:0, 49:1, 49:2, 51:1, 51:2, 52:5, and 53:1 (Table 1). LDI–MS tissue imaging confirmed the overexpression of 10 of these 14 triglycerides in liver tissue cross sections (Figure 1). Moreover, the imaging analyses showed that triglycerides were homogeneously distributed over the whole surface of the liver sections from HFD-fed animals, while they were barely detectable in control animals.

### 3.3. Correlations between Plasma Adipokines and Liver Triglycerides

Consistent with previous reports, compared with the STD-fed mice, the HFD-fed mice had higher plasma concentrations of functionally key adipokines, such as resistin (STD: 1314.33 ± 117.30 pg/mL; HFD: 2756.88 ± 1433.37 pg/mL, *p* < 0.01), leptin (STD: 538.01 ± 139.31 pg/mL; HFD: 5156.59 ± 2262.48 pg/mL, *p* < 0.05), and FABP4 (STD: 282.24 ± 65.82 ng/mL; HFD: 585.31 ± 122.60 ng/mL, *p* < 0.05), and reduced plasma levels of adiponectin (STD: 23.06 ± 3.77 µg/mL; HFD: 11.87 ± 1.27 µg/mL, *p* < 0.05) [31,32]. Given the well-established relationships between several adipokines and NAFLD [19], we assessed the correlations between adipokine plasma levels and the 10 triglyceride species identified in the liver concomitantly by LC–MS and LDI–MS tissue imaging (Table 2). No significant correlation was found between plasma leptin, resistin, or adiponectin and any of the liver triglycerides studied in either STD-fed or HFD-fed animals. Interestingly, plasma FABP4 levels showed strong, significant, and positive correlations with 8 of the 10 identified triglycerides in the HFD group but not in the group of animals fed the STD. Additionally, in the HFD-fed animals, FABP4 was significantly positively correlated with 3 of the 4 triglycerides identified only by LC–MS (Table S1).

Additional multivariate analyses were performed to further explore the associations between circulating FABP4 levels and liver triglyceride composition in HFD-fed mice. The multivariate linear regression model included plasma FABP4, weight, plasma NEFAs, and plasma triglycerides as independent variables and each liver triglyceride as a dependent variable. Confirming the previous analyses, the multivariate analyses identified statistically significant, strong associations between FABP4 plasma concentration and the contents of liver triglycerides (Table 3 and Table S2).

### 3.4. Determination of Plasma FABP4 Origin

Despite that the main source of FABP4 is adipose tissue (for review, see [27,42]), *Fabp4* can also be expressed in livers from NAFLD [43], and has been identified as a predictive factor for poor prognosis [44]. Therefore, in order to explore the origin of plasma FABP4 in our experimental model, we first analyzed the expression of *Fabp4* in livers from both STD- and HFD-fed mice. As it is shown in Figure S4A, diet intervention did not induce changes in the *Fabp4* mRNA levels. Nevertheless, the *Fabp4* mRNA levels from adipose tissue were higher than those from liver (295-fold, *p* < 0.001) (Figure S4B). Accordingly, whereas plasma FABP4 did not correlate with liver *Fabp4* levels (*r* = –0.303, *p* = 0.315), it strongly correlated with adipose tissue *Fabp4* levels (*r* = 0.707, *p* = 0.022).

## 4. Discussion

Excessive fat accumulation in liver is the cornerstone of NAFLD [1,2,3]. Although lipotoxicity associated with abnormal liver lipid composition is considered the main trigger in NAFLD progression, the impacts of different lipid species on this process are not fully understood. Tissue lipid characterization in NAFLD remains a challenge in clinical practice due to the lack of non-invasive methods for its identification and monitoring [6]; therefore, the use of experimental models to explore tissue metabolism alterations is warranted. By employing lipidomic and molecular tissue imaging techniques, we have characterized the main lipid species accumulated in an NAFLD animal model.

Mice fed a diet containing 60% kcal from fat for 12 weeks is a well-established model of the early stages of NAFLD [45], where the liver disorders are not yet permanently established. Our HFD-fed animals showed increased body weight and an altered plasma metabolic profile [31,32] characterized by, among other features, insulin resistance; hypertriglyceridemia; increased levels of resistin, leptin, and FABP4; and reduced levels of adiponectin. Compared with those from STD-fed animals, livers from obese HFD-fed animals showed increased lipid deposition. Approximately 60% of the fat accumulation in NAFLD is derived from exogenous sources [14,15], and the higher VLDL production in this animal model evidenced by high plasma triglycerides and VLDLc is not enough to prevent liver fat accumulation. Additionally, HFD has a direct impact on the fatty acid mitochondrial β-oxidation and the de novo synthesis of fatty acids and triglycerides (for review, see [46,47]). Accordingly, the livers of HFD-fed mice showed altered expression of genes involved in these processes. Endogenous lipogenesis is considered to contribute approximately 25% of the fat accumulation in NAFLD [14,15]. Therefore, both increased fatty acid uptake and production and reduced fatty acid burning may play roles in the ectopic liver fat deposition in NAFLD. While evident fibrosis was not observed in livers from our experimental model, the expressions of *Col1a1* and *Col1a2* were induced in livers from HFD-fed mice, thereby suggesting a transcriptional reprogramming that will lead to liver fibrosis in more advanced stages. Although no significant differences were found, a slight increase was observed in the plasma levels of GOT, GPT, and the GPT/GOT ratio in the HFD-fed mice, suggesting the onset of liver injury. In agreement with this observation, the HFD induced the liver expression of pro-inflammatory genes, thus suggesting the onset of the progression from NAFL to NASH.

The disease progression from NAFL to NASH is related to the accumulation of specific lipotoxic molecules in the liver [8,9,13,48,49,50,51,52,53]. Our untargeted analysis identified as putative triglycerides approximately 51% of the metabolites differentially expressed between the two experimental groups, highlighting the abundance of these molecules. Targeted approaches identified 14 triglycerides upregulated in livers from HFD-fed animals, which highlights them as potential key agents in the development of the disease. Interestingly, 9 of these 14 triglycerides have been identified in previous studies [54,55,56]. Additionally, our study identified 5 new triglycerides upregulated in livers from HFD-fed animals: TG(43:1), TG(44:0), TG(46:0), TG(47:0), and TG(49:0). The differences among studies could be due to the different experimental models used (involving different mice strains and diet compositions) or the different methods for compound identification. These differences highlight the need to standardize methodological approaches to allow comparisons among different laboratories and facilities. Because of the lack of inter-study standardization and the variability among studies, we validated our data by monitoring the spatial distribution of the identified triglycerides by LDI–MS tissue imaging in liver cross sections. Tissue imaging confirmed that 10 of the 14 identified triglycerides were homogeneously distributed over the whole surface of liver sections from HFD-fed animals, while they were barely detectable in control animals.

The identified triglyceride species are mainly composed of long-chain saturated fatty acids, although monounsaturated and polyunsaturated fatty acids were also found in some of them. The fatty acid composition in the identified triglycerides can be determined by the type of diet used. Specifically, butter was used in our HFD to obtain the 60% kcal from fat. Most of the lipid content in this diet comes from long-chain saturated fatty acids (i.e., C16:0 and C18:0), but it also contains both monounsaturated and polyunsaturated fatty acids in lower amounts. Our data agree with those from Puri P et al. that found that both saturated and monounsaturated fatty acids were increased in liver triglycerides from NAFLD patients [57]. Saturated fatty acids have been identified as involved in lipotoxicity mechanisms leading to the evolution of NASH [58]. Given the close relationship between liver and adipose tissue [16,17] and the pivotal roles of fatty acids derived from adipose tissue in NAFLD pathogenesis, we hypothesized that in addition to insulin resistance, adipokines may contribute to the content of specific liver triglycerides in NAFLD. Therefore, we explored the potential correlations between the identified liver triglycerides and the selected adipokines involved in NAFLD pathophysiology, including leptin, resistin, adiponectin [16,17], and FABP4 [29,30]. Interestingly, these adipokines were found to be regulated in plasma from the HFD-fed animals [31,32]. Leptin, resistin, and adiponectin did not correlate with any of the liver triglycerides identified by both LC–MS and LDI–MS tissue imaging. Nevertheless, FABP4 was independently associated with 11 of the 14 triglycerides identified (8 identified by the two methods and 3 identified only by LC–MS) in the animals fed the HFD. No significant associations between FABP4 and any triglycerides were found in the STD-fed animals, thereby highlighting the potential role of FABP4 as a selective biomarker of triglyceride liver composition in NAFLD. Furthermore, multivariate linear regression analysis revealed an influence of FABP4 on liver triglyceride composition. Accordingly, we previously reported that exogenous FABP4 induces intracellular lipid accumulation in HepG2 liver cells [28], which supports the view of FABP4 being an active player in liver metabolic disturbances rather than a mere biomarker of ectopic liver lipid accumulation. Additionally, FABP4 depletion improves NAFLD. Huang-Qi San reduced both plasma FABP4 levels and liver lipid accumulation in HFD-fed rats [59]. Tetrahydrocurcumin decreased lipid accumulation in oleic acid-treated HepG2 cells, potentially by inhibiting the expression of *Fabp4* among other lipogenic proteins [60]. Korean red ginseng improved NASH-related inflammation by reducing both FABP4 mRNA and protein levels [61]. The FABP4 inhibitor BMS309403 ameliorated hepatic steatosis, macrophage infiltration, and cellular ballooning of hepatocytes in mice fed with high-fat, high-cholesterol diet [62]. Therefore, FABP4 inhibition may be considered as a new emerging therapeutic approach for NAFLD.

The molecular mechanisms through which FABP4 may contribute to NAFLD need to be further discussed. Since the main source of circulating FABP4 is adipose tissue (for review, see [27,42]), the mechanisms that regulate its release from adipocytes must be taken into account. Because there are no typical secretory signal peptides in the sequence of FABP4 [42], it is released from adipocytes via non-classical secretion pathways [63,64,65,66]. Specifically, FABP4 is secreted from adipocytes in association with lipolysis regulated by adenylyl cyclase (AC)–protein kinase A (PKA)- and guanylyl cyclase (GC)–protein kinase G (PKG)-mediated signal pathways [67]. Therefore, regulation of these pathways in adipose tissue is a key point involved in the impact of the adipose-derived FABP4 on peripheral tissues, including the liver. Specifically in liver, exogenous FABP4 induces intracellular lipid accumulation in HepG2 liver cells [28]. Nevertheless, the molecular mechanisms of how FABP4 contributes to NAFLD are not fully explored so far. We have previously reported that exogenous FABP4 induces ER stress in HepG2 liver cells [28]. The mechanism by which ER stress impairs insulin actions and metabolic control includes the activation of the c-Jun N-terminal kinase (JNK) through the double-stranded RNA-dependent protein kinase (PKR) and via the inositol-requiring kinase 1 (IRE1) or pancreatic ER kinase (PERK) pathways [68]. In agreement with this, FABP4 induces both IRE1 protein levels and JNK phosphorylation in HepG2 cells [28]. JNK activation has a direct impact on the fatty acid metabolism through downregulation of peroxisome proliferator-activated receptor α (PPARα) [69], and its inhibition increases fatty acid β-oxidation [70]. Therefore, the FABP4-induced fatty acid accumulation in HepG2 cells may be a consequence of the PPARα downregulation induced by JNK activation. On the other hand, pharmacological inhibition of FABP4 by BMS309403 alleviates both acute liver injury and NASH, potentially through JNK and nuclear factor κB (NF-κB) inhibition [62]. Therefore, although further studies are warranted in order to explore additional mechanisms involved in the NAFLD regulation by FABP4, increasing evidence points out that FABP4 may regulate this disease through several mechanisms leading to JNK activation.

## 5. Conclusions

By employing lipidomic and molecular tissue imaging techniques (LC–MS and LDI–MS), we characterized the main lipid molecules accumulated in an NAFLD murine model. Ten triglyceride species were identified by both techniques, and LDI–MS showed a diffuse accumulation pattern. The high percentage of saturated fatty acids in the composition of the identified triglycerides is concordant with the HFD used in the NAFLD model. Despite the finding of a predominantly external acquisition of fat, the transcriptomic data suggest an increase in the de novo lipogenesis mechanisms and a reduction in fatty acid β-oxidation. The identified triglyceride accumulation could not be offset by the higher VLDL production.

FABP4, but not other adipokines, was strongly associated with 8 out of 10 triglycerides found to be upregulated in HFD-fed animals by both metabolomic techniques, further suggesting the exogenous origin of fat accumulated in the liver. Although association data preclude the establishment of any causal effect between plasma FABP4 levels and liver triglyceride composition, our findings strongly support the role of circulating FABP4 as a potential biomarker of NAFLD.

## Figures and Tables

**Table 1:** Liver triglycerides upregulated in high-fat-diet (HFD)-fed mice. The accuracy for the m/z values reported is <0.005 ppm.

				HFD vs. STD	
*m/z*	Triglycerides	Adduct Ion	Formula	Fold Change	*p-*Value
862.7867	TG(51:2)	NH_4_^+^	C_54_H_100_O_6_	7.82	0.004
864.8020	TG(51:1)	NH_4_^+^	C_54_H_102_O_6_	5.40	0.008
836.7710	TG(49:1)	NH_4_^+^	C_52_H_98_O_6_	5.16	0.009
892.8326	TG(53:1)	NH_4_^+^	C_56_H_106_O_6_	4.70	0.006
796.7388	TG(46:0)	NH_4_^+^	C_49_H_94_O_6_	4.14	<0.001
839.7103	TG(49:2)	Na^+^	C_52_H_96_O_6_	3.83	0.001
838.7856	TG(49:0)	NH_4_^+^	C_52_H_100_O_6_	3.59	0.004
771.6474	TG(44:1)	Na^+^	C_47_H_88_O_6_	3.21	0.001
810.7535	TG(47:0)	NH_4_^+^	C_50_H_96_O_6_	3.21	0.007
768.7076	TG(44:0)	NH_4_^+^	C_47_H_90_O_6_	3.04	0.001
752.6759	TG(43:1)	NH_4_^+^	C_46_H_86_O_6_	3.01	0.010
822.7558	TG(48:1)	NH_4_^+^	C_51_H_96_O_6_	2.31	0.003
870.7509	TG(52:5)	NH_4_^+^	C_55_H_96_O_6_	1.98	0.008
824.7706	TG(48:0)	NH_4_^+^	C_51_H_98_O_6_	1.53	0.005

**Table 2:** Correlations of plasma adipokines and liver triglycerides identified by both LC–MS and LDI–MS tissue imaging in both STD- and HFD-fed mice. Data are adjusted for weight, plasma NEFAs, and plasma triglycerides.

	Leptin				Resistin				Adiponectin				FABP4			
	STD		HFD		STD		HFD		STD		HFD		STD		HFD	
	*r*	*p-Value*	*r*	*p-Value*	*r*	*p-Value*	*r*	*p-Value*	*r*	*p-Value*	*r*	*p-Value*	*r*	*p-Value*	*r*	*p-Value*
TG(51:2)	−0.083	0.894	0.071	0.929	0.348	0.566	0.227	0.773	0.100	0.850	−0.669	0.217	0.013	0.980	0.977	0.003
TG(51:1)	−0.255	0.679	0.000	1.000	−0.273	0.656	0.216	0.784	0.039	0.942	−0.695	0.193	−0.169	0.749	0.989	0.011
TG(49:1)	−0.206	0.739	0.049	0.951	−0.148	0.813	0.108	0.892	0.164	0.756	−0.632	0.253	−0.412	0.418	0.984	0.016
TG(53:1)	−0.006	0.992	−0.075	0.925	−0.500	0.391	0.253	0.747	−0.352	0.493	−0.711	0.178	0.121	0.820	0.974	0.026
TG(49:2)	0.377	0.532	0.268	0.732	0.097	0.877	0.166	0.834	0.047	0.930	−0.493	0.399	−0.543	0.266	0.990	0.010
TG(49:0)	0.815	0.093	0.015	0.985	0.308	0.614	0.277	0.723	−0.141	0.791	−0.747	0.147	−0.403	0.428	0.993	0.007
TG(44:1)	0.393	0.513	0.322	0.678	−0.405	0.499	−0.263	0.737	−0.143	0.787	0.112	0.857	−0.296	0.568	0.892	0.108
TG(48:1)	0.434	0.465	0.251	0.749	−0.745	0.148	0.013	0.987	−0.579	0.228	−0.181	0.770	0.594	0.214	0.994	0.006
TG(52:5)	0.188	0.762	0.228	0.772	0.247	0.689	0.264	0.736	0.072	0.893	−0.585	0.301	−0.496	0.317	0.973	0.027
TG(48:0)	0.938	0.018	0.030	0.970	0.257	0.677	0.467	0.533	−0.086	0.871	−0.745	0.149	−0.075	0.888	0.925	0.075

**Table 3:** Associations between plasma FABP4 and liver triglycerides identified by both LC–MS and LDI–MS tissue imaging in the HFD-fed mice. Multiple linear regression results are shown as β-coefficients with CIs and *R^2^* values. Triglycerides are displayed as the dependent variables.

	B (95% CI)	*R^2^*
TG(51:2)	8.26 (6.41 to 10.11)	0.997
TG(51:1)	84.64 (46.60 to 122.69)	0.988
TG(49:1)	68.18 (30.73 to 105.62)	0.985
TG(53:1)	21.52 (6.36 to 36.68)	0.967
TG(49:2)	21.28 (11.86 to 30.70)	0.991
TG(49:0)	6.52 (4.25 to 8.80)	0.990
TG(44:1)	4.59 (−2.49 to 11.68)	0.977
TG(48:1)	101.27 (68.00 to 134.53)	0.996
TG(52:5)	1.72 (0.49 to 2.96)	0.974
TG(48:0)	10.68 (−2.71 to 24.08)	0.882

## Supplementary Materials

The following are available online at https://www.mdpi.com/2218-273X/10/9/1275/s1, **Figure S1:** HFD induces VLDL in C57BL/6J mice. **Figure S2:** HFD induces hepatic steatosis in C57BL/6J mice. **Figure S3:** HFD induces changes in the expression of genes involved in fatty acid intracellular lipid metabolism and inflammation pathways. **Figure S4:** *Fabp4* mRNA levels. **Table S1:** Correlations of plasma adipokines and liver triglycerides identified only by LC-MS in both STD- and HFD-fed mice. **Table S2:** Associations between plasma FABP4 and liver triglycerides identified only by LC-MS in the HFD-fed mice.

## References

[1] Bonora E., Targher G. (2012). Increased risk of cardiovascular disease and chronic kidney disease in NAFLD. Nat. Rev. Gastroenterol. Hepatol..

[2] Wong R.J., Aguilar M., Cheung R., Perumpail R.B., Harrison S.A., Younossi Z.M., Ahmed A. (2015). Nonalcoholic steatohepatitis is the second leading etiology of liver disease among adults awaiting liver transplantation in the United States. Gastroenterology.

[3] Kanwal F., Kramer J.R., Duan Z., Yu X., White D., El-Serag H.B. (2016). Trends in the Burden of Nonalcoholic Fatty Liver Disease in a United States Cohort of Veterans. Clin. Gastroenterol. Hepatol. Off. Clin. Pract. J. Am. Gastroenterol. Assoc..

[4] Lambert J.E., Ramos-Roman M.A., Browning J.D., Parks E.J. (2014). Increased de novo lipogenesis is a distinct characteristic of individuals with nonalcoholic fatty liver disease. Gastroenterology.

[5] Mittal S., El-Serag H.B., Sada Y.H., Kanwal F., Duan Z., Temple S., May S.B., Kramer J.R., Richardson P.A., Davila J.A. (2016). Hepatocellular Carcinoma in the Absence of Cirrhosis in United States Veterans is Associated with Nonalcoholic Fatty Liver Disease. Clin. Gastroenterol. Hepatol. Off. Clin. Pract. J. Am. Gastroenterol. Assoc..

[6] Friedman S.L., Neuschwander-Tetri B.A., Rinella M., Sanyal A.J. (2018). Mechanisms of NAFLD development and therapeutic strategies. Nat. Med..

[7] Titchenell P.M., Lazar M.A., Birnbaum M.J. (2017). Unraveling the Regulation of Hepatic Metabolism by Insulin. Trends Endocrinol. Metab..

[8] Samuel V.T., Shulman G.I. (2016). The pathogenesis of insulin resistance: Integrating signaling pathways and substrate flux. J. Clin. Investig..

[9] Yki-Jarvinen H. (2014). Non-alcoholic fatty liver disease as a cause and a consequence of metabolic syndrome. Lancet Diabetes Endocrinol..

[10] Deacon E.M., Pettitt T.R., Webb P., Cross T., Chahal H., Wakelam M., Lord J.M. (2002). Generation of diacylglycerol molecular species through the cell cycle: A role for 1-stearoyl, 2-arachidonyl glycerol in the activation of nuclear protein kinase C-betaII at G2/M. J. Cell Sci..

[11] Lambertucci F., Arboatti A., Sedlmeier M.G., Motiño O., Alvarez M.D.L., Ceballos M.P., Villar S.R., Roggero E., Monti J.A., Pisani G. (2018). Disruption of tumor necrosis factor alpha receptor 1 signaling accelerates NAFLD progression in mice upon a high-fat diet. J. Nutr. Biochem..

[12] Macrae K., Stretton C., Lipina C., Blachnio-Zabielska A., Baranowski M., Gorski J., Marley A., Hundal H.S. (2013). Defining the role of DAG, mitochondrial function, and lipid deposition in palmitate-induced proinflammatory signaling and its counter-modulation by palmitoleate. J. Lipid Res..

[13] Boslem E., MacIntosh G., Preston A.M., Bartley C., Busch A.K., Fuller M., Laybutt D.R., Meikle P.J., Biden T.J. (2011). A lipidomic screen of palmitate-treated MIN6 beta-cells links sphingolipid metabolites with endoplasmic reticulum (ER) stress and impaired protein trafficking. Biochem. J..

[14] Arguello G., Balboa E., Arrese M., Zanlungo S. (2015). Recent insights on the role of cholesterol in non-alcoholic fatty liver disease. Biochim. Biophys. Acta.

[15] Tamura S., Shimomura I. (2005). Contribution of adipose tissue and de novo lipogenesis to nonalcoholic fatty liver disease. J. Clin. Investig..

[16] Boutari C., Perakakis N., Mantzoros C.S. (2018). Association of Adipokines with Development and Progression of Nonalcoholic Fatty Liver Disease. Endocrinol. Metab..

[17] Divella R., Mazzocca A., Daniele A., Sabba C., Paradiso A. (2019). Obesity, Nonalcoholic Fatty Liver Disease and Adipocytokines Network in Promotion of Cancer. Int. J. Biol. Sci..

[18] Polyzos S.A., Mantzoros C.S. (2015). Leptin in health and disease: Facts and expectations at its twentieth anniversary. Metab. Clin. Exp..

[19] Polyzos S.A., Kountouras J., Zavos C. (2009). Nonalcoholic fatty liver disease: The pathogenetic roles of insulin resistance and adipocytokines. Curr. Mol. Med..

[20] Polyzos S.A., Kountouras J., Zavos C., Tsiaousi E. (2010). The role of adiponectin in the pathogenesis and treatment of non-alcoholic fatty liver disease. Diabetes Obes. Metab..

[21] Hui J.M., Hodge A., Farrell G.C., Kench J.G., Kriketos A., George J. (2004). Beyond insulin resistance in NASH: TNF-alpha or adiponectin?. Hepatology.

[22] Polyzos S.A., Aronis K.N., Kountouras J., Raptis D.D., Vasiloglou M.F., Mantzoros C.S. (2016). Circulating leptin in non-alcoholic fatty liver disease: A systematic review and meta-analysis. Diabetologia.

[23] Zelber-Sagi S., Lotan R., Shlomai A., Webb M., Harrari G., Buch A., Kaluski D.N., Halpern Z., Oren R. (2012). Predictors for incidence and remission of NAFLD in the general population during a seven-year prospective follow-up. J. Hepatol..

[24] Argentou M., Tiniakos D.G., Karanikolas M., Melachrinou M., Makri M.G., Kittas C., Kalfarentzos F. (2009). Adipokine serum levels are related to liver histology in severely obese patients undergoing bariatric surgery. Obes. Surg..

[25] Senates E., Colak Y., Yesil A., Coşkunpinar E., Sahin O., Kahraman O.T., Şenateş B.E., Tuncer I. (2012). Circulating resistin is elevated in patients with non-alcoholic fatty liver disease and is associated with steatosis, portal inflammation, insulin resistance and nonalcoholic steatohepatitis scores. Minerva Med..

[26] Jamali R., Razavizade M., Arj A., Aarabi M.H. (2016). Serum adipokines might predict liver histology findings in non-alcoholic fatty liver disease. World J. Gastroenterol..

[27] Rodriguez-Calvo R., Girona J., Alegret J.M., Bosquet A., Ibarretxe D., Masana L. (2017). Role of the fatty acid-binding protein 4 in heart failure and cardiovascular disease. J. Endocrinol..

[28] Bosquet A., Guaita-Esteruelas S., Saavedra P., Rodríguez-Calvo R., Heras M., Girona J., Masana L. (2016). Exogenous FABP4 induces endoplasmic reticulum stress in HepG2 liver cells. Atherosclerosis.

[29] Koh J.H., Shin Y.G., Nam S.M., Lee M.Y., Chung C.H., Shin J.Y. (2009). Serum adipocyte fatty acid-binding protein levels are associated with nonalcoholic fatty liver disease in type 2 diabetic patients. Diabetes Care.

[30] Kim Y.C., Cho Y.K., Lee W.Y., Kim H.J., Park J.-H., Park D.I., Sohn C.I., Jeon W.K., Kim B.-I., Park S.-E. (2011). Serum adipocyte-specific fatty acid-binding protein is associated with nonalcoholic fatty liver disease in apparently healthy subjects. J. Nutr. Biochem..

[31] Bosquet A., Girona J., Guaita-Esteruelas S., Heras M., Saavedra-Garcia P., Martínez-Micaelo N., Masana L., Rodríguez-Calvo R. (2018). FABP4 inhibitor BMS309403 decreases saturated-fatty-acid-induced endoplasmic reticulum stress-associated inflammation in skeletal muscle by reducing p38 MAPK activation. Biochim. Biophys. Acta Mol. Cell Biol. Lipids.

[32] Rodriguez-Calvo R., Girona J., Rodriguez M., Samino S., Barroso E., De Gonzalo-Calvo D., Guaita-Esteruelas S., Heras M., Van Der Meer R.W., Lamb H.J. (2019). Fatty acid binding protein 4 (FABP4) as a potential biomarker reflecting myocardial lipid storage in type 2 diabetes. Metab. Clin. Exp..

[33] Bligh E.G., Dyer W.J. (1959). A rapid method of total lipid extraction and purification. Can. J. Biochem. Physiol..

[34] Mehlem A., Hagberg C.E., Muhl L., Eriksson U., Falkevall A. (2013). Imaging of neutral lipids by oil red O for analyzing the metabolic status in health and disease. Nat. Protoc..

[35] Fahy E., Sud M., Cotter D., Subramaniam S. (2007). LIPID MAPS online tools for lipid research. Nucleic Acids Res..

[36] Kind T., Liu K.H., Lee D.Y., DeFelice B., Meissen J.K., Fiehn O. (2013). LipidBlast in silico tandem mass spectrometry database for lipid identification. Nat. Methods.

[37] Smith C.A., Want E.J., O’Maille G., Abagyan R., Siuzdak G. (2006). XCMS: Processing mass spectrometry data for metabolite profiling using nonlinear peak alignment, matching, and identification. Anal. Chem..

[38] Vinaixa M., Samino S., Saez I., Duran J., Guinovart J.J., Yanes O. (2012). A Guideline to Univariate Statistical Analysis for LC/MS-Based Untargeted Metabolomics-Derived Data. Metabolites.

[39] Rafols P., Vilalta D., Torres S., Calavia R., Heijs B., McDonnell L.A., Brezmes J., Del Castillo E., Yanes O., Ramírez N. (2018). Assessing the potential of sputtered gold nanolayers in mass spectrometry imaging for metabolomics applications. PLoS ONE.

[40] Rafols P., Castillo E.D., Yanes O., Brezmes J., Correig X. (2018). Novel automated workflow for spectral alignment and mass calibration in MS imaging using a sputtered Ag nanolayer. Anal. Chim. Acta.

[41] Rafols P., Torres S., Ramirez N., Del Castillo E., Yanes O., Brezmes J., Correig X. (2017). rMSI: An R package for MS imaging data handling and visualization. Bioinformatics.

[42] Furuhashi M., Hotamisligil G.S. (2008). Fatty acid-binding proteins: Role in metabolic diseases and potential as drug targets. Nat. Rev. Drug Discov..

[43] Greco D., Kotronen A., Westerbacka J., Puig O., Arkkila P., Kiviluoto T., Laitinen S., Kolak M., Fisher R.M., Hamsten A. (2008). Gene expression in human NAFLD. Am. J. Physiol. Gastrointest. Liver Physiol..

[44] Coilly A., Desterke C., Guettier C., Samuel D., Chiappini F. (2019). FABP4 and MMP9 levels identified as predictive factors for poor prognosis in patients with nonalcoholic fatty liver using data mining approaches and gene expression analysis. Sci. Rep..

[45] Kanuri G., Bergheim I. (2013). In vitro and in vivo models of non-alcoholic fatty liver disease (NAFLD). Int. J. Mol. Sci..

[46] Ferramosca A., Zara V. (2014). Modulation of hepatic steatosis by dietary fatty acids. World J. Gastroenterol..

[47] Simoes I.C.M., Janikiewicz J., Bauer J., Karkucińska-Więckowska A., Kalinowski P., Dobrzyn A., Wolski A., Pronicki M., Zieniewicz K., Dobrzyń P. (2019). Fat and Sugar-A Dangerous Duet. A Comparative Review on Metabolic Remodeling in Rodent Models of Nonalcoholic Fatty Liver Disease. Nutrients.

[48] Perry R.J., Samuel V.T., Petersen K.F., Shulman G.I. (2014). The role of hepatic lipids in hepatic insulin resistance and type 2 diabetes. Nature.

[49] Luukkonen P.K., Zhou Y., Sadevirta S., Leivonen M., Arola J., Orešič M., Hyötyläinen T., Yki-Järvinen H. (2016). Hepatic ceramides dissociate steatosis and insulin resistance in patients with non-alcoholic fatty liver disease. J. Hepatol..

[50] Mauer A.S., Hirsova P., Maiers J.L., Shah V.H., Malhi H. (2017). Inhibition of sphingosine 1-phosphate signaling ameliorates murine nonalcoholic steatohepatitis. Am. J. Physiol. Gastrointest. Liver Physiol..

[51] Hirsova P., Ibrahim S.H., Gores G.J., Malhi H. (2016). Lipotoxic lethal and sublethal stress signaling in hepatocytes: Relevance to NASH pathogenesis. J. Lipid Res..

[52] Han M.S., Park S.Y., Shinzawa K., Kim S., Chung K.W., Lee J.-H., Kwon C.H., Lee K.-W., Lee J.-H., Park C.K. (2008). Lysophosphatidylcholine as a death effector in the lipoapoptosis of hepatocytes. J. Lipid Res..

[53] Ioannou G.N. (2016). The Role of Cholesterol in the Pathogenesis of NASH. Trends Endocrinol. Metab..

[54] Sa R., Zhang W., Ge J., Wei X., Zhou Y., Landzberg D.R., Wang Z., Han X., Chen L., Yin H. (2016). Discovering a critical transition state from nonalcoholic hepatosteatosis to nonalcoholic steatohepatitis by lipidomics and dynamical network biomarkers. J. Mol. Cell Biol..

[55] Tu L.N., Showalter M.R., Cajka T., Fan S., Pillai V.V., Fiehn O., Selvaraj V. (2017). Metabolomic characteristics of cholesterol-induced non-obese nonalcoholic fatty liver disease in mice. Sci. Rep..

[56] Chitraju C., Trotzmuller M., Hartler J., Wolinski H., Thallinger G.G., Lass A., Zechner R., Zimmermann R., Koefeler H., Spener F. (2012). Lipidomic analysis of lipid droplets from murine hepatocytes reveals distinct signatures for nutritional stress. J. Lipid Res..

[57] Puri P., Baillie R.A., Wiest M.M., Mirshahi F., Choudhury J., Cheung O., Sargeant C., Contos M.J., Sanyal A.J. (2007). A lipidomic analysis of nonalcoholic fatty liver disease. Hepatology.

[58] Musso G., Cassader M., Paschetta E., Gambino R. (2018). Bioactive Lipid Species and Metabolic Pathways in Progression and Resolution of Nonalcoholic Steatohepatitis. Gastroenterology.

[59] Li Y., Wang C., Jin Y., Chen H., Cao M., Li W., Luo H., Wu Z. (2020). Huang-Qi San improves glucose and lipid metabolism and exerts protective effects against hepatic steatosis in high fat diet-fed rats. Biomed. Pharmacother. Biomed. Pharmacother..

[60] Chen J.W., Kong Z.L., Tsai M.L., Lo C.Y., Ho C.T., Lai C.S. (2018). Tetrahydrocurcumin ameliorates free fatty acid-induced hepatic steatosis and improves insulin resistance in HepG2 cells. J. Food Drug Anal..

[61] Jeong H., Kim J.W., Yang M.S., Park C., Kim J.H., Lim C.W., Kim B. (2018). Beneficial Effects of Korean Red Ginseng in the Progression of Non-Alcoholic Steatohepatitis via FABP4 Modulation. Am. J. Chin. Med..

[62] Hoo R.L., Lee I.P., Zhou M., Wong J.Y., Hui X., Xu A., Lam K.S.L. (2013). Pharmacological inhibition of adipocyte fatty acid binding protein alleviates both acute liver injury and non-alcoholic steatohepatitis in mice. J. Hepatol..

[63] Xu A., Wang Y., Xu J.Y., Stejskal D., Tam S., Zhang J.-L., Wat N.M., Wong W.K., Lam K.S.L. (2006). Adipocyte fatty acid-binding protein is a plasma biomarker closely associated with obesity and metabolic syndrome. Clin. Chem..

[64] Cao H., Sekiya M., Ertunc M.E., Burak M.F., Mayers J.R., White A., Inouye K., Rickey L.M., Ercal B.C., Furuhashi M. (2013). Adipocyte lipid chaperone AP2 is a secreted adipokine regulating hepatic glucose production. Cell Metab..

[65] Kralisch S., Ebert T., Lossner U., Jessnitzer B., Stumvoll M., Fasshauer M. (2014). Adipocyte fatty acid-binding protein is released from adipocytes by a non-conventional mechanism. Int. J. Obes..

[66] Schlottmann I., Ehrhart-Bornstein M., Wabitsch M., Bornstein S.R., Lamounier-Zepter V. (2014). Calcium-dependent release of adipocyte fatty acid binding protein from human adipocytes. Int. J. Obes..

[67] Mita T., Furuhashi M., Hiramitsu S., Ishii J., Hoshina K., Ishimura S., Fuseya T., Watanabe Y., Tanaka M., Ohno K. (2015). FABP4 is secreted from adipocytes by adenyl cyclase-PKA- and guanylyl cyclase-PKG-dependent lipolytic mechanisms. Obesity.

[68] Fu S., Watkins S.M., Hotamisligil G.S. (2012). The role of endoplasmic reticulum in hepatic lipid homeostasis and stress signaling. Cell Metab..

[69] Drosatos K., Drosatos-Tampakaki Z., Khan R., Homma S., Schulze P.C., Zannis V.I., Goldberg I.J. (2011). Inhibition of c-Jun-N-terminal kinase increases cardiac peroxisome proliferator-activated receptor alpha expression and fatty acid oxidation and prevents lipopolysaccharide-induced heart dysfunction. J. Biol. Chem..

[70] Shen T., Chen X., Li Y., Tang X., Jiang X., Yu C., Zheng Y., Guo H., Ling W. (2017). Interleukin-17A exacerbates high-fat diet-induced hepatic steatosis by inhibiting fatty acid beta-oxidation. Biochim. Biophys. Acta Mol. Basis Dis..

