# Supplementary material for: Hepatic Lipidomics and Molecular Imaging in a Murine Non-Alcoholic Fatty Liver Disease Model: Insights into Molecular Mechanisms
Source: Biomolecules. 2020 Sep 3;10(9):1275. doi: 10.3390/biom10091275 (PMC7563600; doi:10.3390/biom10091275)
Supplement: Supplementary file 1 [file biomolecules-10-01275-s001.pdf]

biomolecules

MDPI

Article

Supplementary Hepatic Lipidomics and Molecular Imaging in a Murine Non-Alcoholic Fatty Liver Disease Model: Insights into Molecular Mechanisms

Ricardo Rodríguez-Calvo [1,2,*], Sara Samino [2,3], Josefa Girona [1,2], Neus Martínez-Micaelo [1,2], Pere Ràfols [2,3], María García-Altares [2,3], Sandra Guaita-Esteruelas [1,2], Alexandra Junza [2,3], Mercedes Heras [1,2], Oscar Yanes [2,3], Xavier Correig [2,3] and Lluis Masana [1,2,*]

1 Vascular Medicine and Metabolism Unit, Research Unit on Lipids and Atherosclerosis, "Sant Joan" University Hospital, Universitat Rovira i Virgili, Institut de Investigació Sanitaria Pere Virgili (IISPV), 43204 Reus, Spain; josefa.girona@urv.cat (J.G.); neus.martinez@urv.cat (N.M.-M.); sandra.guaita@urv.cat (S.G.-E.); mercedes.heras@urv.cat (M.H.)

2 Spanish Biomedical Research Centre in Diabetes and Associated Metabolic Disorders (CIBERDEM), Institute of Health Carlos III, 28029 Madrid, Spain; sara.samino@estudiants.urv.cat (S.S.); pere.rafols@urv.cat (P.R.); maria.garcia-altares@urv.cat (M.G.-A.); alexandra.junza@urv.cat (A.J.); oscar.yanes@urv.cat (O.Y.); xavier.correig@urv.cat (X.C.)

3 Metabolomics Platform, Department of Electronic Engineering (DEEEA), Universitat Rovira i Virgili, 43007 Tarragona, Spain.

* Correspondence: ricardo.rodriguez@ciberdem.org (R.R.-C.); luis.masana@urv.cat (L.M.).

Figure S1

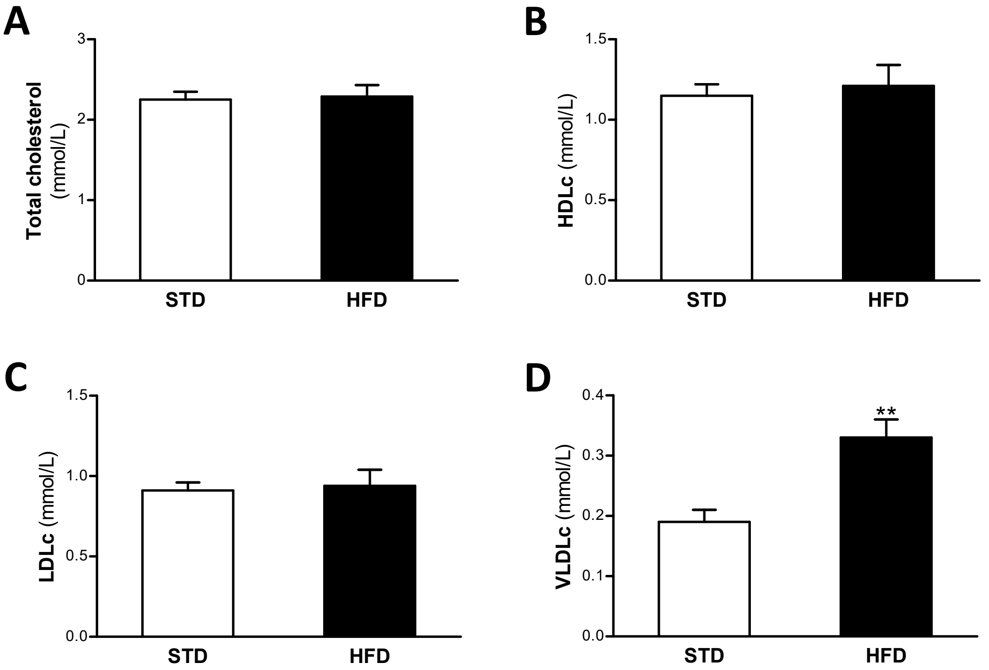

Figure S1. HFD induces VLDL in C57BL/6J mice. Mice were fed a standard (STD) or high-fat diet (HFD) for 12 weeks. After this period, the animals were sacrificed, and plasma samples were collected under fasting conditions. Plasma levels of total cholesterol (**A**), HDLc (**B**), LDLc (**C**) and VLDLc (**D**) are shown in animals fed the STD and those fed the HFD. Data are expressed as the means ± SEM. **$p < 0.01$ *vs* STD-fed mice.

Figure S2

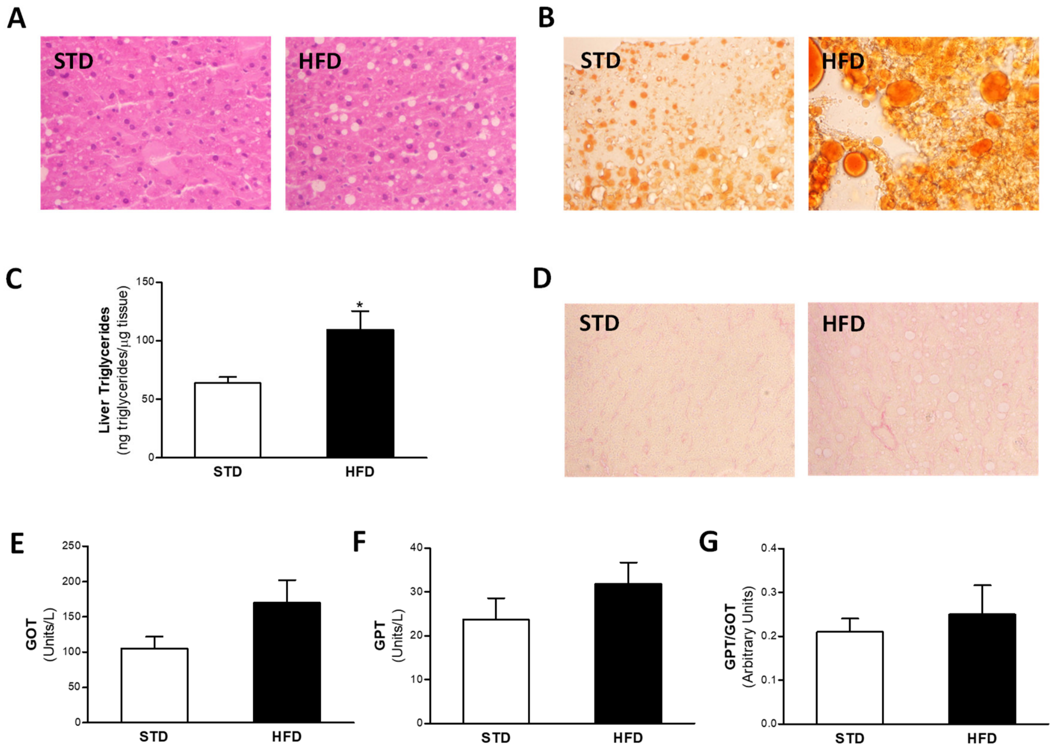

Figure S2. HFD induces hepatic steatosis in C57BL/6J mice. Representative haematoxylin & eosin (**A**) and Oil Red O (**B**) staining, liver triglycerides content (**C**) and Sirius red staining (**D**) in livers from STD- and HFD-fed animals. Plasma levels of GOT (**E**), GPT (**F**) and the GPT/GOT ratio (**G**) in mice fed the STD or the HFD. Images are captured at 400X.

Figure S3

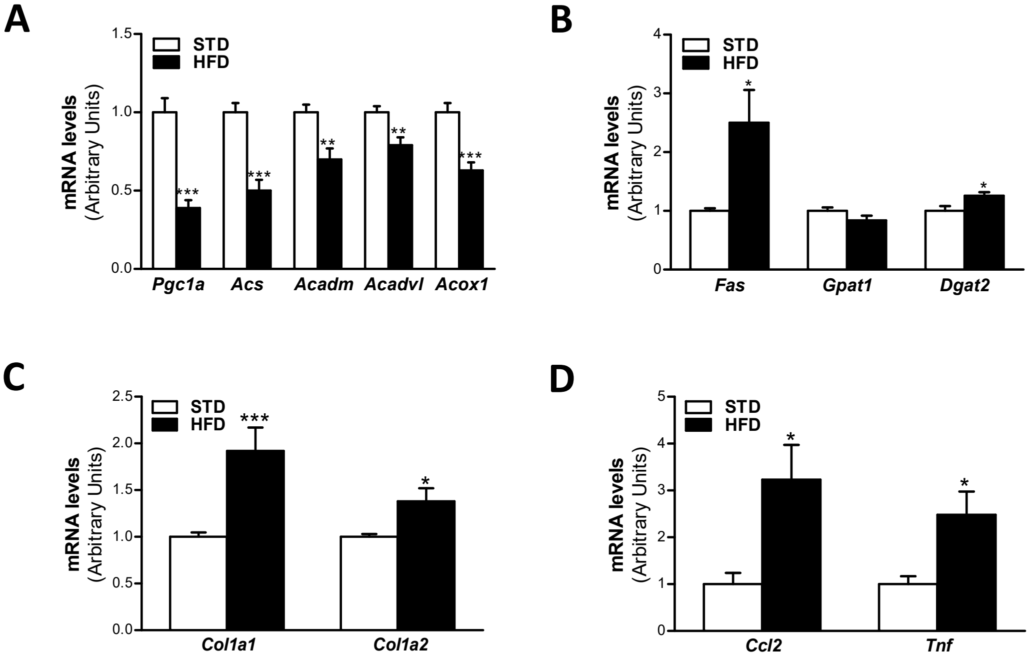

Figure S3. HFD induces changes in the expression of genes involved in fatty acid intracellular lipid metabolism and inflammation pathways. mRNA levels of *Pcg1α*, *Acs*, *Acadm*, *Acadvl*, *Acox1* (**A**), *Fas*, *Gpat1*, *Dgat2* (**B**), *Col1a1*, *Col1a2* (**C**), *Ccl2* and *Tnf* (**D**) in livers from STD- and HFD-fed animals are shown. Data were normalized to the *Tbp* mRNA levels and expressed as the mean ± SEM. (*$p<0.05$; **$p<0.01$; ***$p<0.001$ *vs*. STD-fed animals).

Figure S4

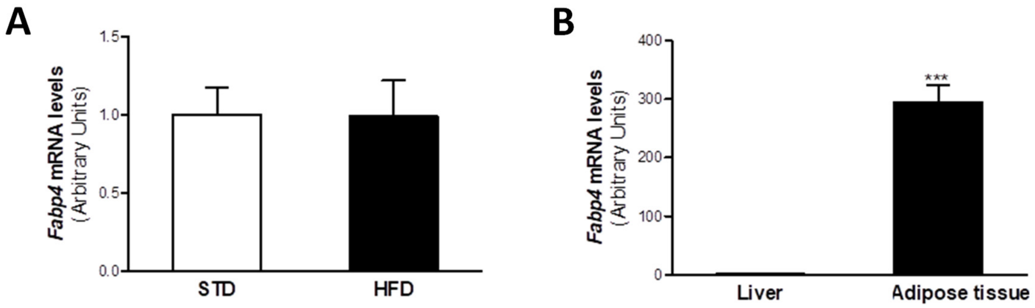

Figure S4. *Fabp4* mRNA levels in livers from STD- and HFD-feed mice (**A**) or in liver and white adipose tissue from HFD-feed mice (**B**) assessed by real-time RT-PCR. The data were normalized to Tbp mRNA levels and are expressed as the mean ± SEM. (****p*<0.001 vs. liver).

Table S1. Correlations of plasma adipokines and liver triglycerides identified only by LC-MS in both STD- and HFD-fed mice. Data are adjusted for weight, plasma NEFAs and plasma triglycerides.

	**Leptin**				**Resistin**				**Adiponectin**				**FABP4**			
	**STD**		**HFD**		**STD**		**HFD**		**STD**		**HFD**		**STD**		**HFD**	
	r	***p-value***	***r***	***p-value***	***r***	***p-value***	***r***	***p-value***	***r***	***p-value***	***r***	***p-value***	***r***	***p-value***	***r***	***p-value***
TG(46:0)	0.547	0.340	0.059	0.941	-0.430	0.470	0.153	0.847	-0.458	0.361	-0.021	0.973	0.545	0.263	**0.966**	**0.034**
TG(47:0)	0.430	0.469	0.023	0.977	0.439	0.459	0.253	0.747	0.147	0.781	-0.710	0.179	-0.586	0.222	**0.994**	**0.006**
TG(44:0)	0.865	0.058	0.116	0.884	-0.147	0.813	-0.766	0.234	-0.377	0.461	0.375	0.534	0.210	0.690	0.360	0.640
TG(43:1)	0.322	0.598	0.263	0.737	-0.070	0.911	-0.092	0.908	0.067	0.899	-0.476	0.417	-0.592	0.216	**0.974**	**0.026**

Table S2. Associations between plasma FABP4 and liver triglycerides identified only by LC-MS in the HFD-fed mice. Multiple linear regression results are shown as β-coefficients with CIs and R^2 values. Triglycerides are displayed as the dependent variables.

	**B (95% CI)**	R^2
TG(46:0)	3.29 (0.60 to 5.98)	0.979
TG(47:0)	4.56 (3.06 to 6.06)	0.993
TG(44:0)	1.69 (-11.61 to 14.98)	0.800
TG(43:1)	0.53 (0.16 to 0.90)	0.986
